# Opioid prescribing for acute postoperative pain: an overview of systematic reviews related to two consensus statements relevant at patient, prescriber, system and public health levels

**DOI:** 10.1186/s12871-023-02243-5

**Published:** 2023-08-30

**Authors:** C. L. McCorquodale, R. Greening, R. Tulloch, P. Forget

**Affiliations:** 1 University of Aberdeen School of Medicine, Medical Sciences and Nutrition, Aberdeen, Scotland, UK; 2 NHS Greater Glasgow and Clyde, Glasgow, Scotland, UK; 3 Department of Anaesthetics, NHS Grampian, Aberdeen, Scotland, UK; 4 Pain AND Opioid After Surgery (PANDOS) European Society of Anaesthesiology and Intensive Care (ESAIC) Research Group, Brussels, Belgium

**Keywords:** Opioid, Acute pain, Postsurgical, Postoperative

## Abstract

**Background:**

National guidelines for rational opioid prescribing for acute postoperative pain are needed to optimise postoperative pain control and function whilst minimising opioid-related harm.

**Objectives:**

This overview of systematic reviews aims to summarise and critically assess the quality of systematic reviews related to the 20 recommendations from two previously published consensus guideline papers (ten relevant at patient and prescriber levels and ten at a system / Public Health level). It also aims to identify gaps in research that require further efforts to fill these in order to augment the evidence behind creating national guidelines for rational opioid prescribing for acute postoperative pain.

**Methods:**

A systematic database search using PubMed/MEDLINE and Cochrane was conducted in November 2022. Furthermore, reference lists were reviewed. All identified systematic reviews were assessed for eligibility. Data from each study was extracted using a pre-standardised data extraction form. The methodological quality of the included reviews was assessed by two independent reviewers using the AMSTAR 2 checklist. Descriptive synthesis of the results was performed.

**Results:**

A total of 12 papers were eligible for analysis. Only eight out of the total 20 prioritised recommendations had systematic reviews that provided evidence related to them. These systematic reviews were most commonly of critically low quality.

**Conclusion:**

The consensus papers provide guidance and recommendations based on the consensus of expert opinion that is based on the best available evidence. However, there is a lack of evidence supporting many of these consensus statements. Efforts to further analyse interventions that aim to reduce the rates of opioid prescribing and their adverse effects should therefore continue.

## Introduction

### Background

Between the years of 1998 and 2018, opioid prescribing has more than doubled in England [1]. Liberal prescribing of opioids for postoperative pain relief increases the risk of persistent postoperative opioid use and adverse outcomes, which has reached epidemic proportions in certain countries. These adverse outcomes include dependence, addiction, opioid use disorders, opioid-induced ventilatory impairment and overdose-related deaths [2]. A study by Gomes et al*.* assessed the burden of opioid-related deaths in the USA and found that the percentage of all deaths attributable to opioids increased by an alarming 292% (from 0.4% to 1.5%) between 2001 and 2016 [3]. Prescribers may unwittingly be playing a major part in this epidemic. A cohort study in 2019 found that 76.2% of surgical patients in the USA filled an opioid prescription within the first 7 days after a surgical procedure compared to 11.1% of surgical patients in Sweden [4]. An iatrogenic driving factor to the overprescribing of opioids was the unrestricted titration of opioids to numerical pain scores, such as the ‘Pain as the 5^th^ Vital Sign’ campaign, which has now been discredited [5].

To solve this problem, guidelines have been proposed in different countries, as well as consensus documents to implement their content, but also to consider aspects that have not included. In the United Kingdom (UK), the Faculty of Pain Medicine released recommendations composed by a multi-organisational and multidisciplinary collaboration, setting out guiding principles for preoperative, perioperative, postoperative and post discharge opioid management. The aim of these recommendations is to reduce postoperative opioid use and the adverse effects caused by them. In summary, the Best Practice document gives the following recommendations on aspects of postoperative opioid management: 1) pain relief should be optimised; 2) pain assessment should involve functional assessment; 3) immediate-release opioids are preferred; 4) give advice on medicine self-administration on discharge; 5) local protocols for the prescription of discharge medications after surgery should be developed; 6) hospital discharge letter must explicitly state the recommended opioid dose, amount supplied and planned duration of use; 7) identify patients for de-escalation of opioids; 8) guidance should be given about necessary medicine review post-discharge [6].

But guidelines are necessary but not enough, and local guidance should consider local aspects and include educational programme, as recently considered as the highest priority in the context of multimodal, opioid-sparing, analgesia [7]. Accordingly, there have been two consensus statements published in the UK since 2020, not only relevant at patient and prescriber levels, but also at system and Public Health levels, aiming at facilitating local implementation and education. The first consensus paper, by Levy et al*.*, was an international multidisciplinary consensus statement, which aimed to provide guidance in order to “assist healthcare professionals and hospitals across the world to implement effective opioid stewardship practices that achieve a balance between the administration of sufficient opioid analgesia to facilitate recovery and restoration of function, while concurrently minimising the risk of opioid-related harms” [8]. Levy et al*.* provides ten priority recommendations based on best evidence and, in the absence of such, expert opinion. The second statement was by Forget et al*.*, which aimed to propose a consensus, not only on the prescribing of opioids, but also on policies for system-level interventions. Their ten recommendations were approved by a panel of experts in the field, along with healthcare representatives from different related medical disciplines and patient representatives from around the world. Therefore, the research reflects the view of a multi-stakeholder panel and represents a breadth of perspectives [2].

Although both papers provide valuable recommendations for opioid prescribing practices, they are based on expert opinion. Expert opinion is often sought during the development of governance and regulatory policies when there is insufficient empirical evidence to implement a policy or change [9], but it is essential now, in the context of evidence-based medicine, centred around the incorporation of knowledge gained through clinical trials, systematic reviews (SRs) and meta-analyses [10] to reappraise the quality of the underlying evidence and to identify knowledge gaps.

## Aims

The primary aim of this overview of SRs is to summarise the evidence and critically assess the quality of SRs that are relevant to the ten priorities of each of the Levy et al. [8] and Forget et al [2] consensus statement papers. By doing so, this overview will assess the quality of evidence supporting these two consensuses. The secondary aim is to identify gaps in research that require further efforts to fill these in order to augment the evidence behind the creation of a consensus statement for rational opioid prescribing for acute postoperative pain.

## Methods

### Review design

This overview of reviews was conducted in accordance with the Cochrane Handbook for the Systematic Review of Interventions and reported following the Reporting guideline for overviews of healthcare interventions: the Preferred Reporting Items for Overviews of Reviews (PRIOR) statement [11, 12].

Due to the nature of this literature-based project, no ethics approval was required. However, each of the SRs included state the ethics considerations and approval that they required.

### Eligibility criteria

The inclusion and exclusion criteria are shown in Table 1.

**Table 1:** Summary of inclusion and exclusion criteria. This table summarises the inclusion and exclusion criteria applied to assess study eligibility during this overview

*Study Criteria*	*Inclusion Criteria*	*Exclusion Criteria*
*Study design*	Systematic review (± meta-analysis)	Non-reviews; protocols
*Population*	Postsurgical patients exposed to acute / sub-acute pain (adults ± children)	Non-postsurgical patients; patients exposed to chronic pain; paediatric-only patients
*Intervention*	An intervention related to one of either the ten priorities described by Levy et al. *(2020)* [8] or ten priorities described by Forget et al. *(2022)* [2]	Non-relevant intervention
*Comparison*	A method to analyse the efficacy of the intervention	No method to analyse the efficacy of the intervention
*Outcome*	An outcome related to acute postoperative pain / opioid use / relevant clinical outcomes	An outcome unrelated to acute postoperative pain / opioid use / relevant clinical outcomes

### Search strategy

The search strategy involved looking at the SRs cited in reference lists of the consensus statements by Levy et al. [8] and Forget et al. [2] in addition to an electronic literature search, which was conducted during November 2022 using PubMed/MEDLINE. Various search strategies were used to identify SRs relating to the ten priorities of the Levy et al*. *(2020) consensus and the ten priorities of the Forget et al*.* consensus [2]. The complete search strategies are reported at the end of this paper under “Search Terms for Database Search”. A search limit of ‘systematic review’ and ‘meta-analysis’ was added. Papers were retrieved through additional sources, such as hand-searching the reference list of the Acute Pain Management document [13] and consulting an expert in the field for relevant papers. Backward snowballing was also used to identify relevant papers missed through the database search strategy.

### Study selection

All identified citations were collated and uploaded to RefWorks 2 and duplicates were removed. Two independent reviewers (CM, RT) screened the titles and abstracts of identified articles to assess relevancy. Irrelevant articles were removed at this stage. Subsequently, full texts of selected articles were accessed and further screened by the same two reviewers (CM, RT) by applying the inclusion and exclusion criteria to assess eligibility. Articles that did not meet the eligibility criteria were removed. Any queries or uncertainties were discussed and resolved through discussion and consensus between the two reviewers (CM, RT) and a supervisor (PF).

### Date extraction

A standardised data extraction form was predefined to portray the study characteristics of included articles. The following data was extracted from each article into this data extraction form by a single author (CM): author, title, year of publication, country, search period, number of primary studies included, total number of participants, effect size, intervention, outcome measures, study methods, quality evaluation method, degree of certainty, meta-analysis (yes/no) and main findings.

### Quality assessment

The methodological quality of the included SRs was assessed by two independent reviewers (CM, RG) using the Assessment of Multiple Systematic Reviews 2 (AMSTAR 2) checklist [14]. The AMSTAR 2 checklist consists of ten items, including seven critical items. AMSTAR 2 does not generate an overall ‘score’, but rather it categorises the quality of the assessed article as one of the following: high (≤ 1 non-critical item weakness); moderate (> 1 non-critical item weakness); low (1 critical item weakness, with or without non-critical weaknesses); critically low (> 1 critical item weakness, with or without non-critical weaknesses). Each reviewer entered their score for each item of the checklist for each SR into an Excel document. Any discrepancies were discussed between the reviewers until consensus was reached.

### Data synthesis

A quantitative meta-analytic synthesis of the included SRs was not performed due to great heterogeneity across the reviews arising from differences in sample characteristics, as well as differences between methods and outcome measures. Therefore, the data was grouped to the relevant ten priorities of the Levy consensus and the ten priorities of the Forget consensus and descriptive synthesis of the results was performed [2, 8].

## Results

### Search outcomes

**Fig. 1:**
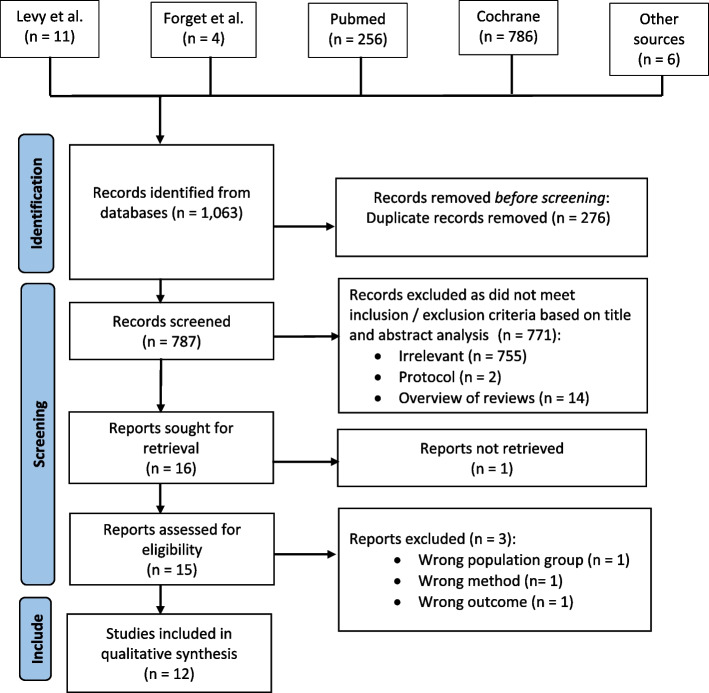
Preferred Reporting Items for Systematic Reviews and Meta-Analyses (PRISMA) flow diagram (2020). This diagram illustrates the selection process of studies included in this systematic review and is based on the 2020 PRISMA flow diagram [15]

In total, 1,063 studies were identified. 276 duplicate records were removed, leaving 787 studies to be screened. After title and abstract analysis, a further 771 papers were excluded based on inclusion and exclusion criteria. The full texts of 16 studies were sought for retrieval. One study could not be fully retrieved, therefore 15 full texts were retrieved and assessed. 12 studies met the inclusion and exclusion criteria and were used in this overview (Fig. 1). The list of studies excluded after full-text analysis and reasons are shown at the end of this paper.

### Study characteristics

A summary of the detailed characteristics of the included SRs is presented in Table 2 and their findings are summarised in Table 3. All 12 included SRs were published between 2016 and 2022. The included authors are from the USA (*n* = 5) [16–20], UK (*n* = 2) [21, 22], Australia (*n* = 1) [23], Canada (*n* = 1) [24], France (*n* = 1) [25], Poland (*n* = 1) [26] and Switzerland (*n* = 1) [27]. Three reviews included only randomised controlled trials (RCTs) [22, 25, 27]; three other reviews included only non-randomised studies of intervention (NRSIs) [19, 23, 24]; five reviews included both RCTs and NRSIs [16–18, 20, 21]; and one review did not specify the type of studies it included [26]. The number of studies in the SRs ranged from 6 to 135 with a mean of 41.33, and the total number of participants in these studies ranged from 810 to 1,922,743 with a mean of 220,197.58. However, one study did not report the total number of participants [17]. Only four out of the 12 included SRs were meta-analysed [19, 22, 26, 27].

**Table 2:** The characteristics of included systematic reviews. This table describes the characteristics of each study that is included in this overview. The author, year of publication, country, number of primary studies, number of participants, search period, intervention, primary outcome measures and quality evaluation method of each review are all stated

*Authors*	Year	Country	Included study design	No. of studies *(total no. of participants)*	Search period	Intervention	Primary outcome measures	Quality evaluation method
*Albrecht *et al*. * [27]	2019	Switzerland	RCTs	27 *(1,630)*	Up to 30 June 2019	High-dose vs low-dose intraoperative opioids	Pain score at rest at 24 postoperative hours	GRADE
*Arwi & Schug * [23]	2020	Australia	NRSIs	28 *(661,441)*	Up to 1 December 2018	Opioids prescribed at discharge after inpatient care	The potential for harm of discharge opioids including excessive prescribing of discharge opioids, improper storage and disposal of opioids	Newcastle–Ottawa Quality Assessment Scale
*Baamer *et al*.* [21]	2022	UK	RCTs and NRSIs	31 *(12,498)*	Up to August 2020	Unidimensional and functional assessment tools used for postoperative patients	Measurement error, cross-cultural validity, reliability, responsiveness, and hypothesis testing for construct validity	Modified version of the Newcastle–Ottawa Quality Assessment Scale; and COSMIN criteria for methodological quality
*Bicket *et al*. * [16]	2017	USA	RCTs and NRSIs	6 *(810)*	Up to 20 July 2016	Opioids prescribed for acute postoperative pain	The number of patients reporting any unused opioids	Newcastle–Ottawa Quality Assessment Scale
*Feinberg *et al*. * [24]	2018	Canada	NRSIs	11 *(3,562)*	Up to 17 December 2016	Opioids prescribed for acute postoperative pain	The quantity of opioid medication used post-discharge	None
*Horn *et al*. * [17]	2020	USA	RCTs and NRSIs	43 *(Not reported)*	Not reported	Preoperative psychoeducational methods	The quality of preoperative psychoeducation and its effects on the outcome of surgery	Oxford levels of evidence
*Lamplot *et al*. * [18]	2021	USA	RCTs and NRSIs	16 *(3,077)*	Up to 2 October 2019	Opioids prescribed for acute postoperative pain	Rates of disposal of unused opioids and the reported disposal mechanisms for unused opioids	SIGN checklists for cohort studies and RCTs
*Lawal *et al*. * [19]	2020	USA	NRSIs	33 *(1,922,743)*	Up to 30 June 2019	Postoperative opioid use	Risk factors associated with prolonged opioid use after surgery	Newcastle–Ottawa Quality Assessment Scale
*Martinez *et al*. * [25]	2017	France	RCTs	135 *(13,287)*	Up to August 2015	Non-opioid analgesics and tramadol prescribed for acute postoperative pain	Morphine consumption, pain, incidence of nausea, vomiting at 24 h and severe adverse effects	Cochrane Risk of Bias tool
*Powell *et al*. * [22]	2016	UK	RCTs	105 *(10,302)*	Up to May 2014	Psychological preparation in adults undergoing elective surgery under general anaesthetic	Postoperative outcomes including pain, behavioural recovery, length of stay and negative affect	Cochrane Risk of Bias tool
*Sobol-Kwapinska *et al*. * [26]	2016	Poland	Not reported	53 *(10,749)*	January 1960 – 30 November 2015	Preoperative psychological factors	Acute postoperative pain and analgesic consumption	Quality in Prognostic Studies tool
*Wetzel *et al*. * [20]	2018	USA	RCTs and NRIs	8 *(2,272)*	January 2000 – March 2018	Behavioural intervention associated with postoperative prescribing	Postoperative opioid prescribing	Quality Assessment Tool for Quantitative Studies

*USA* United States of America, *UK* United Kingdom, *RCTs* Randomised controlled trials, *NRSIs* Non-randomised studies of intervention, *GRADE* Grading of Recommendations, Assessment, Development and Evaluations, *SIGN* Scottish Intercollegiate Guidelines Network

**Table 3:** Overview of the findings of included systematic reviews. This table summarised the findings of included systematic review. The main findings, meta-analysis and effect size (if reported) and degree of certainty of each review are all stated. In addition, the priorities of the Levy et al. and Forget et al. consensuses that the reviews relate to have been reported [2, 8]

*Authors*	Main findings	Meta-analysis (yes/no)	Effect size with [95% confidence interval]	Degree of certainty	Priority
*Albrecht *et al	There is low certainty of evidence that high-dose intraoperative opioid administration increases pain scores in the post-operative period when compared with a low-dose regimen	Yes	Mean difference: -0.22 [-0.39, -0.05]	Low	Forget et al*.* 4
*Arwi & Schug*	The current discharge opioid prescribing practices can be improved. Lack of patient education regarding storage and disposal of opioids also contributes to the increasing rate of opioid misuse, diversion, and unintended long-term use. More high-quality research with comparable outcomes is needed. Evidence-based hospital guidelines and public health policies are needed to improve opioid stewardship	No	Not reported	Good – poor	Levy et al*.* 6 + 10
*Baamer *et al	This review found no evidence that any one unidimensional tool has superior measurement properties in assessing postoperative pain. In addition, because promoting function is a crucial perioperative goal, psychometric validation studies of functional pain assessment tools are needed to improve pain assessment and management	No	Not applicable	High – very low	Levy et al*.* 3
*Bicket *et al	Post-operative prescription opioids often go unused, unlocked, and undisposed, suggesting an important reservoir of opioids contributing to non-medical use of these products	No	Not reported	Intermediate	Levy et al*.* 10
*Feinberg *et al	Surgical patients are using substantially less opioids that prescribed. There is a lack of awareness regarding proper disposal of leftover medication, leaving excess opioids that may be used inappropriately by the patient or others. Education for providers and clinical practice guidelines that provide guidance on prescription of outpatient of opioids are required	No	Not reported	Not reported	Levy et al*.* 6
*Horn *et al	By addressing the psychological needs of patients through preoperative education, one can decrease postoperative recovery time and postsurgical acute pain. Reduced postsurgical acute pain results in fewer opioid prescriptions, which theoretically lowers the patient’s risk of developing chronic postsurgical pain, and potentially offers a novel concept using pre-emptive pain psychoeducation as a part of multimodal pain management solution to the opioid epidemic	No	Not reported	1a – 3b	Levy et al*.* 2
*Lamplot *et al	Opioid pain medications are overprescribed postoperatively, and baseline rates of surplus opioid disposal are low. While it remains unclear whether patient education alone increases rates of safe opioid disposal, drug disposal kits or bags do appear to significantly increase these rates	No	Not reported	Acceptable	Levy et al*.* 10
*Lawal et a*	In this study, preoperative use of opioids and cocaine and the presence of comorbid pain conditions before surgery had the strongest associations with prolonged opioid use after surgery. These largely modifiable patient-level risk factors may be included as part of a comprehensive strategy to screen for at-risk individuals requiring transition to non-opioid interventions after surgery while ensuring appropriate short-term opioid use to manage postoperative pain. Research is needed to further investigate the association between surgical pain and prolonged opioid use after surgery	Yes	Not applicable	High	Levy et al*.* 1
*Martinez *et al	A combination of acetaminophen with either an NSAID or nefopam was superior to most non-morphine analgesic used alone, in reducing morphine consumption. Efficacy was best with three non-morphine analgesic used alone (α-2 agonists, NSAIDs and COX-2 inhibitors) and least with tramadol and acetaminophen. There is insufficient trial data reporting adverse events	No	Morphine consumption: -1 [-83 to 6.3] to -23.9 [-40.1 to -7.7] Pain: 0.8 [-14.9 to 16.5] to -12.4 [-21 to -3.8]	High – low and unclear risk of bias	Levy et al*.* 4
*Powell *et al	The evidence suggested that psychological preparation may be beneficial for the outcomes postoperative pain, behavioural recovery, negative affect and length of stay, and is unlikely to be harmful. However, at present, the strength of evidence is insufficient to reach firm conclusions on the role of psychological preparation for surgery. Further analyses are needed to explore the heterogeneity in the data, to identify more specifically when intervention techniques are of benefit. As the current evidence quality is low or very low, there is a need for well‐conducted and clearly reported research	Yes	Not reported	Low – very risk of bias	Levy et al*.* 2
*Sobol-Kwapinska *et al	Significant preoperative psychological correlates of acute postsurgical pain were the following: pain catastrophizing, expectation of pain, anxiety (state and trait), depression, optimism, negative affect and neuroticism/psychological vulnerability. Results of meta-analyses suggested that pain catastrophizing was most strongly associated with acute postsurgical pain. It must be noted that the expression ‘the most common/frequent correlates’ should not be confused with the ‘most important correlates’	Yes	Correlation: *r* = 0.24 [0.11 to 0.36] to 0.41 [0.28 to 0.52]	Moderate – low risk of bias	Levy et al*.* 2
*Wetzel *et al	In this systematic review, interventions operating at a physician or organizational level (e.g., workflow changes) have shown positive results, while interventions at the patient level (e.g., patient education) have shown mixed results. Monitoring for negative consequences was key across the studies evaluated. The studies reviewed provide evidence that clinician-mediated and organizational-level interventions are powerful tools in creating change in postsurgical opioid prescribing. This summary highlights paucity of high-quality studies that provide clear evidence on the most effective intervention at reducing postoperative opioid prescribing	No	Not reported	Low	Forget et al*.* 1

There was heterogeneity with regards to quality evaluation methods used in the included SRs. Three SRs used the Newcastle–Ottawa Quality Assessment Scale [16, 19, 23]; two SRs used the Cochrane Risk of Bias Tool [22, 25]; one SR used a modified version of the Newcastle–Ottawa Quality Assessment scale and Consensus-based Standards for the selection of health Measurement Instruments (COSMIN) criteria [21]; one SR used Grading of Recommendations, Assessment, Development and Evaluations (GRADE) [27]; one SR used the Oxford levels of evidence [17]; one SR used the Scottish Intercollegiate Guidelines Network (SIGN) checklists for cohort studies and RCTs [18]; one SR used the Quality in Prognostic Studies tool [26]; one SR used the Quality Assessment Tool for Quantitative Studies [20]; and, finally, one SR did not use a quality evaluation method to assess the quality of its included studies [24]*.*

### Quality of the evidence

The quality of the included SRs was assessed using AMSTAR 2 checklist, which is presented in Table 4. The supplementary of three papers could not be accessed [18, 20, 25]. The authors of these SRs were contacted to request access to their supplementary material; however, we did not receive a response. The SRs by Lamplot et al*.* and Martinez et al*.* could, therefore, not be fully assessed using the AMSTAR 2 checklist [18, 25]. The SR by Wetzel et al*.* had enough information in its full text and therefore was fully assessed; however, we cannot say if their supplementary material contains information that may alter their AMSTAR 2 tool results [20].

**Table 4:** Quality assessment of included systematic reviews. This table demonstrates the quality of each systematic review using the AMSTAR 2 checklist [14]. The supplementary material of two papers could not be accessed [18, 25]. Therefore, a full quality appraisal could not be done on these systematic reviews

	Research question and inclusion criteria include PICO components	A priori design	Justification of included study designs	Comprehensive literature search strategy	Study selection performed in duplicate	Data extraction in duplicate	List of excluded studies with justifications	Included studies describes in adequate detail	Satisfactory technique to assess risk of bias	Report on funding sources in studies	Appropriate method for statistical combination	Impact of RoB on meta-analysis results	Account for RoB in individual studies when interpreting results	Explanation of heterogeneity in results	Assessed publication bias	Reported conflicts of interest	**Overall Quality**
*Albrecht *et al	Y	Y	Y	PY	Y	Y	N	P	Y	N	Y	Y	Y	Y	Y	Y	**Low**
*Arwi & Schug*	Y	N	Y	N	Y	Y	N	Y	Y	N	N	N	Y	N	N	Y	**Critically low**
*Baamer *et al	Y	N	Y	P	Y	Y	Y	Y	Y	N	N	N	Y	Y	N	Y	**Low**
*Bicket *et al	Y	N	Y	P	Y	Y	N	Y	Y	Y	N	N	N	Y	N	Y	**Critically low**
*Feinberg *et al	Y	N	Y	P	Y	N	N	Y	N	N	N	N	N	N	N	N	**Critically low**
*Horn *et al	N	N	Y	Y	Y	N	N	N	N	N	N	N	N	N	N	N	**Critically low**
*Lamplot *et al	Y	N	Y		Y	Y		Y		N	N	N	Y	Y	N	Y	
*Lawal et a*	Y	Y	Y	P	Y	Y	N	Y	N	N	Y	Y	Y	Y	Y	Y	**Critically low**
*Martinez *et al	Y	Y	Y	P	Y	Y			Y	Y	Y	Y	N	Y	N	Y	
*Powell *et al	Y	Y	Y	Y	Y	Y	Y	Y	Y	N	Y	Y	Y	Y	Y	Y	**High**
*Sobol-Kwapinska *et al	Y	N	N	P	Y	Y	N	N	Y	N	Y	Y	Y	Y	Y	Y	**Critically low**
*Wetzel *et al	Y	N	Y	P	N	Y	N	P	N	N	N	N	N	N	N	Y	**Critically low**

*RoB* Risk of bias*, Y* Yes*, P* Partial yes*, N* no

According to the criteria of AMSTAR 2, seven of the ten SRs that could be fully assessed were of critically low quality, two were of low quality and one was of high quality. Items 2, 7 10, 11, 12 and 15 were rated particularly low amongst the included SRs. Only one SR that was able to be fully assessed reported the funding sources of the included SRs [16]. A summary of the 20 recommendations domains, and the presence or not of SRs and their respective degree of certainty is presented in Table 5.

**Table 5:** Summary of Findings. This table presents a summary of the findings of this overview of SRs. It summarises the 20 recommendations of the Levy et al. (2020) [8] and the Forget et al. (2022) [2] consensus statements, states the presence or not of relevant SRs and/or meta-analyses, and the respective degree of certainty (“Uncertain” in the absence of SR)

*Recommendation domain*	SRs/meta-analysis quality	Degree of certainty
***Levy *et al.*’s (2020)*** [8] ***priorities***		
*Risks with opioids*	1 SR with MA (critically low quality)	Low
*Preoperative optimisation*	2 SRs with MA + 1 SR without MA (high – critically low quality)	High – low
*Functional outcomes-based analgesia*	1 SR without MA (low quality)	Low
*Multimodal analgesia*	1 SR without MA (quality could not be assessed)	Uncertain
*Long-acting opioids*	No SRs	Uncertain
*Patient-centred treatment duration*	2 SRs without MA (critically low quality)	Low
*Post-discharge repeat prescriptions*	No SRs	Uncertain
*Opioid-induced ventilatory impairment*	No SRs	Uncertain
*Modifiable factors*	No SRs	Uncertain
*Safe opioid storage and disposal*	3 SRs without MA (critically low quality	Low
***Forget *et al.*’s (2022)*** [2] ***priorities***		
*Opioid Stewardship Steering Committee*	1 SR without MA (critically low quality)	Low
*Safe and accountable opioid use policies*	No SRs	Uncertain
*Policies on opioid prescriptions determinants*	No SRs	Uncertain
*Opioid treatment (dose and duration) policies*	1 SR with MA (low quality)	Low
*Follow-up and referral guidelines*	No SRs	Uncertain
*Monitoring of opioid prescriptions*	No SRs	Uncertain
*Preventing obstacles to access appropriate opioid prescription*	No SRs	Uncertain
*Opioid disposal*	No SRs	Uncertain
*Benchmarking*	No SRs	Uncertain
*Improved interaction primary/secondary care*	No SRs	Uncertain

*SR Systematic review*

## Discussion

### Main findings

The aim of this overview was to summarise the evidence and critically assess the quality of SRs that are relevant to the ten priorities of the Levy consensus and the ten priorities of the Forget consensus [2, 8]. This overview has identified a total of 12 SRs, which related to only six out of the ten priorities of the Levy consensus and two of the ten priorities of the Forget consensus. This means that a total of 12 priorities from both consensuses do not have evidence that could be identified through the methods of this overview that either supports or opposes them. Thus, we have identified a gap in research that requires further attention and efforts to fill to enhance stewardship of opioid prescribing for acute postoperative pain.

The SRs that were identified were generally of low quality according to the AMSTAR 2 checklist (seven were critically low, two were low, one was high and two could not be fully appraised). Hence, further research is required to produce evidence of a higher quality to support the consensuses and pave the way for future safer opioid prescribing. The AMSTAR 2 tool was developed in 2017 as an upgraded version of the original AMSTAR tool. It is a well-used valid and reliable appraisal tool [28].

### Implications of findings within current literature

The prescribing of opioids for acute postoperative pain remains a highly controversial topic. The two consensus statements provide very promising progress for the development of national protocols for the safe prescribing and stewardship of postoperative opioids. With regards to these two consensus statements, both at patient/prescriber and system/Public Health levels, they are based on variable levels of certainty and on analyses of variable quality. This has implications when integrating these aspects into clinical practice. Indeed, levels of certainty can impact both guidelines and guidance, even if both can be based on other sources, at the condition that generalisable, high quality, evidence, is identifiable. Expert opinion can then be considered when evidence is lacking or impossible to generate. Differentiating these levels of evidence is essential to robustly secure high quality local guidance and educational programmes, which have been described as essential to implement recommendations and to confirm their local validity [7]*.* Sng et al. graded education as the highest priority that determines the use of opioid-sparing analgesia. Their recommendation is that more leadership and specific guidelines for multimodal analgesia could increase the adoption of these techniques. Our work could inform that kind of efforts and, here, follow specific suggestions that could be considered for implementation, in regard to the level of certainty and quality.

### Specific suggestions for improvement

With regards to priority 1 of the Levy consensus (“all patients undergoing surgery should be assumed to be at risk of developing persistent postoperative opioid use and opioid-induced ventilatory impairment and may need interventions to mitigate those risks”) [8], the SR by Lawal et al*.* provided evidence to support this statement. They concluded that strategies, such as proactively screening for at-risk individuals, should be priorities to reduce the substantial burden that persistent opioid use after surgery elicits on public health. Lawal et al*.* reported that preoperative use of opioids and cocaine and the presence of comorbid pain conditions before surgery were found to have the strongest associations with persistent opioid use after surgery [19]. These modifiable risk factors could be included in a comprehensive approach to identify patients at higher risk of persistent opioid use and opioid-induced ventilatory impairment. However, it should be noted that this evidence was of critically low quality according to the AMSTAR 2 checklist.

Priority 2 of the Levy consensus (“Consider optimising management of pre-operative pain and psychological risk-factors before surgery, including weaning of opioids where possible. Ensure realistic expectations of postoperative pain control, both in hospital and after discharge”) [8] has three included SRs that provide evidence to support it. First of all, the SR by Horn et al*.* concluded that addressing the psychological needs of patients through preoperative education can decrease acute postoperative pain, and therefore decrease the need for opioid consumption [17]. Additionally, the SR by Powell et al*.* found evidence that suggested preoperative psychological preparation may be beneficial for various outcomes, such as postoperative pain, behavioural recovery, negative affect and length of stay in hospital [22]. However, the strength of evidence they found was insufficient, thus they recommended that further research is required to support this. Finally, the SR by Sobol-Kwapinska et al*.* analysed the relations between presurgical psychological factors and acute postoperative pain. They identified numerous psychological variables that could be considered for optimising preoperative psychological risk factors before surgery, as recommended by Levy et al.  [8, 26]. The quality of evidence according to the AMSTAR 2 checklist was noted to be critically low for the SRs by Horn et al*.* and Sobol-Kwapinska et al*.*, but was high for the Powell et al*.* SR [17, 22, 26].

Baamer et al*.* provided evidence for priority 3 of the Levy consensus (“provision of opioid analgesia should be guided by functional outcomes, rather than unidimensional pain scores alone”) [8] by challenging the validity and reliability of unidimensional tools to quantify acute postoperative pain. They also discovered that studies on functional outcomes assessment tools were scarce, and therefore proposed more research is necessary to assess the validity and reliability of such tools [21]. The quality of this SR was low, according to the AMSTAR 2 tool. Thus, future research of a higher quality could be beneficial to further support priority 3 of the Levy consensus.

Priority 4 of the Levy consensus (“multimodal analgesia should be optimised and patients educated about the use of non-pharmacological and non-opioid analgesia to reduce the amount and duration of opioids required to restore function”) [8] was supported through evidence from the SR by Martinez et al. This paper concluded that a multimodal regimen of non-opioid analgesics was superior to solitary use of a single non-opioid analgesia in reducing acute postoperative pain and morphine consumption [25]. The supplementary material of this SR was unavailable, resulting in full quality appraisal being unachievable. More research could be done to further assess multimodal analgesic regimens to increase the validity of this recommendation from Levy et al.

There are two SRs found through the methodology of this overview that provide evidence for the 6^th^ priority of the Levy consensus (“a patient-centred approach should be used to limit the number of tablets and the duration of usual discharge opioid prescriptions, typically to less than a week”) [8]. Arwi and Schug suggest that the current opioid prescribing practices could be improved. The studies they analysed showed that discharge opioids contribute to prolonged opioid use [23]. However, more high-quality research with comparable outcomes is needed. Additionally, the SR by Feinberg et al*.* reported that surgical patients are using substantially less opioid than prescribed, leading to excess opioids that may be used inappropriately by patients or others. The authors agreed that strategies and clinical practice guidelines are needed to better educate prescribers and help standardise postoperative opioid prescriptions [24]. It should be noted that both these SRs were of critically low quality according to the AMSTAR 2 tool. It would be beneficial for research of a higher quality be carried out to further support the Levy consensus.

The 10^th^ priority of the Levy consensus (“patients should be advised on safe storage and disposal of unused opioids and directed to avoid opioid diversion to other individuals (e.g. sharing with friends and family)”) [8] was also supported by the Arwi and Schug SR. This paper reported that a lack of patient education regarding safe storage and disposal of opioids contributes to the increasing rate of opioid misuse, diversion and unintended persistent opioid use. However, the authors recommend that more high-quality research is needed on this topic [23]. The SR by Bicket et al*.* provides further evidence for the 10^th^ priority. This paper concluded that postoperative opioid prescriptions often go unused, unlocked and undisposed, leading to a reservoir of opioids that contribute to the non-medical use of these products [16]. Although both these SRs are of critically low quality according to the AMSTAR 2 checklist, they still provide important evidence that supports the 10^th^ priority of the Levy consensus.

The SR by Lamplot et al*.* provides further evidence for priority 10 of the Levy consensus. They found that opioids are overprescribed for acute postoperative pain, and baseline rates of surplus opioid disposal are low. Furthermore, their results showed that drug disposal kits or bags help to significantly increase these rates [18]. Due to the supplementary material being unavailable, we could not fully assess the quality of this SR. However, it provides valuable evidence for future strategies to increase the safe disposal of unused opioids.

With regards to the Forget consensus, the 1^st^ priority (“the presence of a Pain Management, Analgesia or Opioid Stewardship Steering Committee, with multidisciplinary representation from Key Stakeholders is a priority in the context of acute pain, especially in the hospital”) [2] has one included SR that provides supporting evidence. Their results showed evidence that clinician-mediated and organisation-level interventions are effective at reducing postoperative opioid prescribing [20]. The quality of this SR was critically low. However, it provides useful evidence to aid the development of an evidence-based clinical practice guidelines.

Finally, Albrecht et al*.* found that there is overall low certainty of evidence that high-dose intraoperative opioids in patients under general anaesthesia increases pain scores and contributes to hyperalgesia in the postoperative period when compared to low-dose regimen. However, they proposed that additional robust methodology trials could better define the impact of each opioid regime on hospital and health-system recourses [27]. This agrees with priority 4 of the Forget consensus (“policies should be developed providing guidelines on maximum doses and duration of treatment for high-risk medications such as opioids and high-risk combinations”) [2] by suggesting more trials should be undertaken in order to help develop such policies. The quality of the Albrecht et al*.* SR was low according to the AMSTAR 2 checklist.

### Implications of findings for future research

The number of drug-related deaths has vastly risen over the past few decades in the UK. According to the National Drug-Related Deaths Database (NDRDD) for Scotland, there were 1,209 deaths in 2018 that were drug related in Scotland. Opioids were implicated in 77% of these deaths. This is a significant increase from 2017, when there were 867 drug-related deaths in Scotland [29]. In England and Wales, there were 3,756 drug-related deaths in 2018, a 16% increase from 2017 [30]. The rise in drug-related deaths is thought to be due to the increased availability and misuse of prescription and illicit opioids due to irrational prescribing, amongst other factors. There are concerns that the UK is closely following the trends of the devastating opioid epidemic seen in the USA. A solution to the contributing factor of liberal opioid prescribing for acute postoperative pain could be the implementation of national guidance and protocols.

The Levy and Forget consensuses provide a strong framework for such protocols. They are predominantly expert opinion based [2, 8]. Historically, medicine was based on the consensus of experts and their opinions on best practices. Though expert opinion is a highly regarded and useful method of gathering information, it is more valid when used concomitant with evidence-based literature for the creation of healthcare policies and protocols [9]. Further research is required to provide evidence of a higher quality to support these consensus statements.

### Strengths & limitations

This overview included SRs of varying settings that covered a range of topics regarding rational opioid prescribing, enabling the concise evaluation and summarisation of literature related to the ten priorities of the Levy consensus and the ten priorities of the Forget consensus [2, 8]. It therefore offers valuable insight into the evidence behind the two consensuses that are predominantly based on expert opinion. Furthermore, this overview was conducted in accordance with the Cochrane Handbook for the Systematic Review of Interventions, which is well-known and well-used guidance, thus increasing reliability [11].

There are several limitations of this overview. Firstly, there was one SR that could not be accessed for full-text analysis which may have offered valuable evidence [31]. Additionally, the supplementary material of three included SRs was not available, despite requesting access from the authors, resulting in full quality appraisals being incomplete [18, 20, 25].

There was significant heterogeneity amongst the SRs regarding interventions, outcome measures, and quality evaluation method, with only four out of the 12 included SRs including meta-analysis. This meant that the SRs were not comparable. However, they provided valuable evidence for the aim of this overview.

The search strategy aimed to identify SRs for evidence for the ten priorities of the Levy consensus and the ten priorities of the Forget consensus through various database searches [2, 8]. However, predefined search strategies cannot be solely relied upon as it is probable that these various searches may have failed to identify all available relevant SRs. Backward snowballing was used to identify potential missed SRs. Finally, another limitation of this overview is that the included papers were from a wide range of countries. Though this may provide useful information that could shape future rational opioid prescribing protocols, it may not be applicable to UK guidance.

## Conclusion

In conclusion, this overview of SRs provides valuable insight into the evidence behind the Levy et al*.* and Forget et al*.* consensus statements on rational opioid prescribing. However, there is a dearth of research that is required to implement valid and reliable nation opioid prescribing protocols. This overview found that there are not enough papers with high quality evidence to support the Levy et al*.* and Forget et al*.* consensus statements. The papers that were identified were mainly of low quality. Therefore, more research of a higher quality is required. The liberal prescribing of opioids for acute postoperative pain requires urgent attention. For now, it could be greatly beneficial to implement the recommendations given in the Levy et al*.* and Forget et al*.* consensus statements. The consensus papers provide guidance based on the consensus of expert opinion that is based on the best available evidence. However, efforts to further analyse interventions that aim to promote safter opioid prescribing and reduce their adverse effects should continue.

## Search terms for database search

1. ((wean opioids) OR (taper opioids)) AND (acute pain) AND ((postsurg*) OR (postop*)).
  Results = 24
2. ((preoperative education) OR (opioid education)) AND (acute pain) AND ((postsurg*) OR postop*)).
  Results = 20
3. (unidimensional pain score) AND ((postsurg*) OR (postop*)).
  Results = 2
4. (abnormal pain trajectory) AND (acute pain) AND ((postsurg*) OR (postop*)).
  Results = 0
5. ((non-opioid) OR (opioid-free) AND (acute pain) AND ((postsurg*) OR (postop*)).
  Results = 12
6. (long-acting opioids) AND (acute pain) AND ((postsurg*) OR (postop*)).
  Results = 2
7. (compound opioids) AND (acute pain) AND ((postsurg*) OR (postop*)).
  Results = 1
8. (multimodal analgesia) AND (acute pain) AND ((postsurg*) OR (postop*)).
  Results = 30
9. ((weaning opioids) OR (tapering opioids)) AND (acute pain) AND ((postsurg*) OR (postop*)).
  Results = 1
10. (repeat prescription) AND (acute pain) AND ((postsurg*) OR (postop*)).
  Results = 0
11. ((persistent pain) OR (chronic pain) AND (acute pain) AND ((postsurg*) OR (postop*)).
  Results = 190

## Data Availability

The datasets used and/or analysed during the current study available from the corresponding author on reasonable request.
